# Effect of cognitive training on cortisol levels in patients with neurocognitive disorders

**DOI:** 10.1093/geronb/gbaf243

**Published:** 2026-01-08

**Authors:** Marina De Rui, Giulia Salerno Trapella, Chiara Ceolin, Filippo Ceccato, Giorgia Antonelli, Adele Ravelli, Rita Andreuzza, Enrico Conti, Michela Sarlo, Alessandra Coin, Bruno Micael Zanforlini, Anna Bertocco, Chiara Curreri, Irene Tizianel, Daniela Mapelli, Giuseppe Sergi, Maria Devita

**Affiliations:** Department of Medicine (DIMED), University of Padua, Padua, Italy; Department of Medicine (DIMED), University of Padua, Padua, Italy; Department of Medicine (DIMED), University of Padua, Padua, Italy; Department of Neurobiology, Care Sciences and Society, Karolinska Institutet and Stockholm University, Aging Research Center, Stockholm, Sweden; Endocrinology Unit, Department of Medicine (DIMED), University of Padua, Padua, Italy; Endocrine Disease Unit, University of Padua, Padua, Italy; Laboratory Medicine, Department of Medicine (DIMED), University of Padua, Padua, Italy; Department of Medicine (DIMED), University of Padua, Padua, Italy; Department of Medicine (DIMED), University of Padua, Padua, Italy; Neurology Clinic, Hospital of Santorso, Vicenza, Italy; Department of Communication Sciences, Humanities and International Studies, University of Urbino Carlo Bo, Urbino, Italy; Department of Medicine (DIMED), University of Padua, Padua, Italy; Department of Medicine (DIMED), University of Padua, Padua, Italy; Department of Medicine (DIMED), University of Padua, Padua, Italy; Department of Medicine (DIMED), University of Padua, Padua, Italy; Endocrinology Unit, Department of Medicine (DIMED), University of Padua, Padua, Italy; Endocrine Disease Unit, University of Padua, Padua, Italy; Department of General Psychology (DPG), University of Padua, Padua, Italy; Department of Medicine (DIMED), University of Padua, Padua, Italy; Department of Medicine (DIMED), University of Padua, Padua, Italy; Department of General Psychology (DPG), University of Padua, Padua, Italy; (Psychological Sciences Section)

**Keywords:** Alzheimer’s disease, Mild cognitive impairment, Cognitive stimulation

## Abstract

**Objectives:**

Elevated cortisol levels are linked to a greater risk and faster progression of neurocognitive disorders (NCDs). While interventions such as exercise and mindfulness have shown benefits in reducing cortisol, the impact of cognitive training (CT) on cortisol regulation remains unexplored. This study investigated whether CT affects cortisol levels and secretion patterns in individuals with minor or major NCD and compared its effects with those of pharmacological treatment.

**Methods:**

Sixty-two older adults with NCD and 43 healthy controls were recruited from the University Hospital of Padua in Italy. Among patients with NCD, 34 underwent CT (CT-NCD group), and 28 received pharmacological treatment (PH-NCD group). Salivary cortisol was measured at six points during the day, at baseline, and at 3 months (T1) and 6 months (T2) post-intervention.

**Results:**

Compared with pharmacological treatment (PH), CT showed a larger percentage decrease of daily cortisol exposure area under the curve (AUC) from baseline; however, the between-group difference did not remain statistically significant after covariate adjustment, and the only robust time-point effect was in the afternoon (*F*(1,47)=5.13; *p* = .028). Morning values decreased within groups, but between-group differences in the CAR were not significant; at bedtime, CT showed only a trend towards lower cortisol than PH (*p* = .071). Median morning values changed from 7.75 to 6.20 in CT and from 5.80 to 5.15 in PH.

**Discussion:**

Cognitive training may help lower cortisol levels and enhance cognitive function in NCD patients, suggesting its potential as a nonpharmacological tool to modulate hypothalamic–pituitary–adrenal axis activity. Larger randomized studies are needed to confirm and extend these findings.

## Graphical abstract

**Graphical Abstract:**
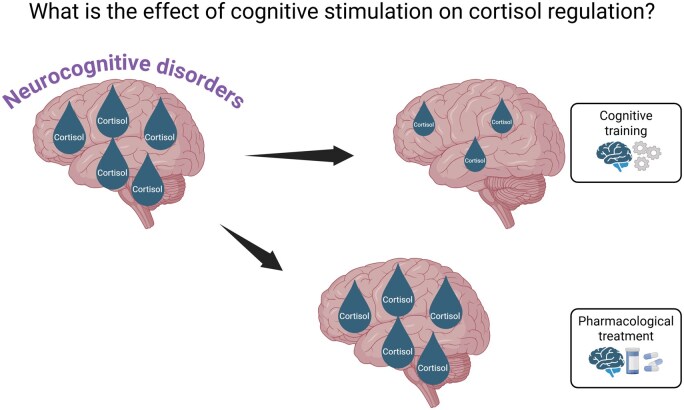

Cortisol secretion is regulated by the hypothalamic-pituitary-adrenal (HPA) axis, a complex neuroendocrine system that responds to physical and psychological stress stimuli. The process begins with the production of corticotropin releasing hormone (CRH) by the paraventricular nucleus of the hypothalamus (PVN), which in turn stimulates the release of adrenocorticotropic hormone (ACTH), stimulating the secretion of cortisol from the adrenal cortex (Chan & Debono, 2010; Lightman et al., 2020; Milligan Armstrong et al., 2021; Robertson-Dixon et al., 2023; Rusch et al., 2023). The pattern of cortisol secretion follows a circadian rhythm, with a positive peak upon awakening (cortisol awakening response [CAR]) and a subsequent diurnal decrease, reaching the lowest levels in the evening (Adam et al., 2017; Clow & Smyth, 2020; Dziurkowska & Wesolowski, 2021). Cortisol has important regulatory effects, influencing arousal, energy and metabolic processes; immune and inflammatory system functioning; and cognition and mood behavior (Sapolsky et al., 2000). Alterations in cortisol levels or in its rhythm can have consequences for health outcomes (Bride et al., 2021), as evidenced by previous studies that have revealed associations between alterations in cortisol rhythm and mental and physical health disorders (Doane et al., 2013; Heim et al., 2008; Matthews et al., 2006). Growing evidence supports a significant link between elevated cortisol levels and increased risk and faster progression of cognitive decline (­Csernansky et al., 2006; Cuadrado-Tejedor et al., 2012), since cortisol induces hippocampal damage through impairment of neurogenesis and induction of neurodegeneration, in addition to increasing beta-amyloid production and decreasing its clearance (Dronse et al., 2023; Ouanes & Popp, 2019; Yeram et al., 2021).

These data, underlining how elevated cortisol levels contribute to cognitive decline and neurodegeneration, suggest that approaches that have proven effective in reducing cortisol levels might represent a promising strategy to mitigate these deleterious effects on the brain.

Accordingly, activities such as aerobic exercise (Smyth et al., 2019), dance (Ho et al., 2018; Predovan et al., 2019), mindfulness (Flook et al., 2013; Goldberg et al., 2018), and art and music therapy (Bleibel et al., 2023; Kaimal et al., 2016) have been shown to reduce cortisol levels and increase the volume of the hippocampal regions and prefrontal areas. Cognitive training (CT), which consists of involving people affected by neurocognitive disorders (NCDs) in activities aimed at improving general cognitive and social functioning (Devita et al., 2021), is a nonpharmacological treatment that is also recommended by the National Institute for Health and Care Excellence (NICE) (Wang et al., 2018). Emerging evidence suggests that cognitive interventions may reduce allostatic load and support HPA axis regulation, with reported benefits on cortisol levels, cognitive performance, and emotional well-being (Da Silva et al., 2021; Manigault et al., 2019). To the best of our knowledge, no study has explored whether the clinical improvement obtained through CT is associated with a reduction in cortisol levels and/or an improvement in its secretion pattern.

The present study aimed to investigate the trends in salivary cortisol levels and circadian patterns in patients with minor and mild major NCD before and after CT and to compare the effects of CT and pharmacological treatment. It should be noted that the group assignment was not random. In accordance with clinical recommendations, patients with milder impairment (MCI or mild NCD) were preferentially assigned to cognitive training, whereas those with more advanced impairment were treated with pharmacological therapy. Thus, the CT and PH groups differed at baseline in cognitive status.

## Methods

Participants were enrolled from 2022 to June 2024 among those attending an outpatient visit to the Cognitive Disorders and Dementia Center (CDCD) of the Geriatrics Unit at the University Hospital of Padova in Italy. For the study, the following inclusion criteria were considered: age ≥ 65 years; diagnosis of minor (mild cognitive impairment—MCI) or major neurocognitive disorder (NCD), which was defined according to DSM-5 criteria, which include evidence of significant cognitive decline in one or more cognitive domains (e.g. memory, executive function) that interferes with independence in daily activities; informed consent to participate in the study.

The exclusion criteria included hospitalization, chronic inflammatory diseases, a diagnosis of major depression or other interfering psychiatric pathologies, ongoing steroid or immunomodulatory therapy, and an altered sleep–wake rhythm. Participants who were receiving both cognitive training and pharmacological therapy were also excluded.

A control group of 43 cognitively healthy participants aged ≥65 years was included for comparison of cortisol values. The same inclusion and exclusion criteria were applied to the control group.

All participants with a neurocognitive disorder were accompanied by a caregiver. The caregiver did not participate directly in the study but was responsible for ensuring the correct saliva collection using Salivette, returning the collected samples, and supporting adherence to both pharmacological and non-pharmacological treatments.

All the participants signed informed consent forms and were informed of their right to withdraw from the study at any time. This study was approved by the Ethical Committee of the University of Padova (Protocol n. 15228) in accordance with the Declaration of Helsinki.

### Group-specific interventions for cognitive decline

The choice of treatment (cognitive stimulation vs. pharmacological therapy) was determined by the outpatient specialist who evaluated the patient, in accordance with current Italian and international guidelines. Specifically, cognitive training was administered to patients with MCI or mild forms of major NCD, in accordance with clinical guidelines, as pharmacological treatment is not recommended for MCI. Additionally, the cognitive training group included patients with mild major NCD for whom pharmacological therapy was contraindicated due to clinical conditions such as bradycardia or atrioventricular block (AVB). Cognitive training is a nonpharmacological, evidence-based intervention designed to improve the cognitive function of individuals with cognitive impairment. Specifically, it is a structured tool that stimulates different cognitive areas, such as memory, attention, language, and executive functions, and focuses on the social context of small groups or single sessions (Woods et al., 2023). Patients in the CT-NCD group underwent a cycle of cognitive training therapy, consisting of two individual sessions per week of 50 min each, for a total duration of three months (a total of 24 sessions). To avoid potential sources of bias, the CT was administered by highly trained staff. Each session, conducted entirely in a quiet and cozy room specifically equipped for this aim at the dedicated offices of the University Hospital of Padua, started with the reality orientation therapy (ROT), namely the patient’s orientation within three domains: personal, spatial, and temporal. Then, the session proceeded with structured stimulation consisting of pencil-and-paper exercises specific to each of the five cognitive domains assessed: memory, language, spatial and temporal orientation, attention, and logic. The pencil-and-paper exercises were taken from “Dementia. 100 Exercises,” the most well-known Italian book for cognitive training (Bergamaschi et al., 2007). Exercises were selected for each cognitive domain, and the degree of difficulty was fixed weekly, starting at a low level and becoming progressively more difficult. The same number of pencil-and-paper exercises were selected for each cognitive domain, so that all cognitive functions were equally stimulated.

Pharmacological treatment was otherwise reserved for patients with major NCD, based on clinical severity: acetylcholinesterase inhibitors (donepezil 5–10 mg/day or rivastigmine 4.6–9.5 mg/day) were administered to patients with mild-to-moderate Alzheimer’s disease, while memantine 10–20 mg/day was used in moderate-to-severe cases. The treatment duration aligned with the study timeline (3 months), and caregivers helped ensure adherence. No treatment withdrawals occurred due to side effects.

### Collected data

Each participant underwent the following tests/assessments:

- The medical history data collected included personal data (sex, age, date of birth), physiological and pharmacological history, and remote pathological history data (a list of comorbidities and severity scores from the Cumulative Illness Rating Scale Comorbidity and Severity Indexes [CIRS-CI and CIRS-SI, respectively; Linn et al., 1968]);
- Evaluation of cognitive performance with the Mini-Mental State Examination (MMSE) (Folstein et al., 1975);
- The levels and pattern of salivary secretion of cortisol were determined via Salivette, which is a device containing a cotton roll (Sarstedt, Numbrecht, Germany), according to the manufacturer’s instructions. A set of six Salivette devices was used for saliva collection. The participants (and caregivers) were asked to choose a standard day for collecting saliva samples at well-defined times: upon waking, two hours after waking, before lunch, two hours after lunch, before dinner, and before sleeping. The measurement of salivary cortisol in six samples was performed in all subjects at baseline (T0), i.e. before the start of cognitive training (for the CT-NCD group) or pharmacological therapy (for the PH-NCD group), and after three months (T1); moreover, for the CT-NCD group, the measurement was also performed 3 months after the end of cognitive training (T2). Salivary cortisol was measured by liquid chromatography-tandem mass spectrometry (LC–MS/MS), a method that was previously validated (Antonelli et al., 2015). Salivary cortisol was analyzed at the Laboratory Medicine Unit in collaboration with the Endocrinology Unit of the University Hospital of Padua.

Cortisol assays were performed by laboratory personnel blinded to the treatment group. MMSE assessments were conducted in a clinical context by personnel aware of group assignment.

### Statistical analyses

Continuous quantitative variables are expressed as the mean ± SD or as the median (interquartile range). The normal distribution of continuous variables was assessed via the Shapiro–Wilk test. To compare variables between groups, quantitative variables were analyzed via the Mann–Whitney and Kruskal–Wallis tests, with *p* values adjusted for MMSE scores in group comparisons. Correlations were evaluated via Pearson’s correlation coefficient (r) for normally distributed variables and Spearman’s rank correlation coefficient (rs) for nonnormally distributed variables. For between-group post-intervention differences, we fitted ANCOVA models that included baseline cortisol, age, sex, education, MMSE, and CIRS; adjusted *p*-values reported in tables refer to these models. The area under the curve (AUC) was calculated using the trapezoidal rule, which approximates the integral of the cortisol measurements over time. This method involves summing the areas of consecutive trapezoids formed by the cortisol values at different time points. The AUC provides an estimate of overall cortisol exposure and was computed separately for different groups. A two-tailed *p*-value <.05 was considered statistically significant in all analyses. All statistical analyses were performed via SPSS software version 29.

## Results

### Cortisol levels in subjects with NCD and controls

**Figure 1.**
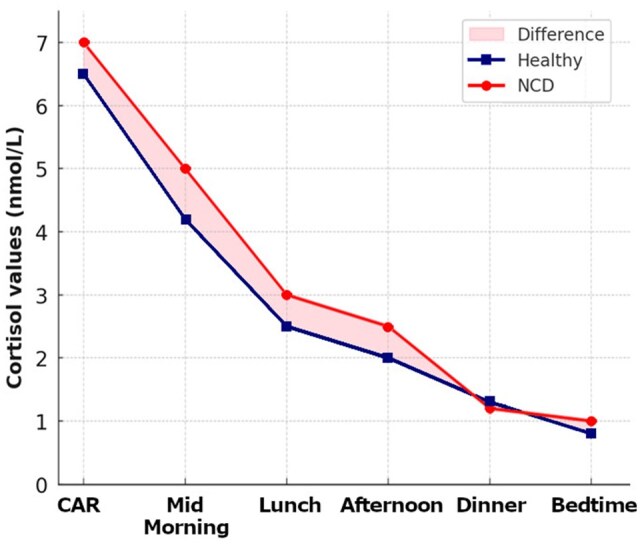
Cortisol patterns in subjects with NCD and in the control group. CAR = Cortisol Awakening Response; NCD = neurocognitive disorder.

The sample consisted of 62 subjects with neurocognitive disorders (52 with major NCD and 10 with minor NCD), with a mean age of 80.9 ± 5.5 years (36 female). Among patients with major NCD, 27 were diagnosed with mild NCD (20 in the CT group and 7 in the PH group), while 25 had moderate NCD (4 in the CT group and 21 in the PH group). The control group, the mean age was 72.6 ± 5.8 years, and 26 patients were female. The circadian pattern (Figure 1) was maintained in both groups, with higher values in the morning than in the evening. Based on AUC, the NCD group maintained higher cortisol levels throughout the day than the healthy group, with cumulative exposure greater than 16% over 24 hr.

The general characteristics of the patients in the control group and in the CT-NCD and PH-NCD groups are reported in Table 1. Among the enrolled subjects with NCD, 34 underwent CT, and 28 underwent pharmacological therapy. The two groups were comparable in terms of age, comorbidities, and baseline cortisol values. As expected, they differed significantly in global cognitive status, with lower MMSE scores in the PH-NCD group (20.8 ± 4.6 vs. 24.3 ± 2.6, *p* < .001).

**Table 1.** Baseline characteristics of the control group and of the two groups of patients with neurocognitive disorders (NCDs) undergoing cognitive training (CT-NCD) or pharmacological therapy (PH-NCD).

Variable	Control group (*n *= 43)	CT-NCD (*n *= 34)	PH-NCD (*n *= 28)	*p* (unadjusted)	*p* (adjusted)
**Age (years)**	72.6 ± 5.8	80.0 ± 5.1	82.2 ± 5.7	.119	.245
**Education (years)**	7.8 ± 2.9	10.4 ± 5.2	7.3 ± 4.9	.021	.145
**MMSE score**	30.0 ± 0.0	24.3 ± 2.6	20.8 ± 4.6	<.001	–
**CIRS-CI**	1.5 (1.0–3.0)	1.4 (0.2;2.8)	2.0 (1.0;3.0)	.553	.915
**Salivary cortisol (nmol/L)**					
**CAR**	6.6 (3.7;11.4)	7.8 (5.2;9.8)	6.5 (4.0;8.9)	.232	.030
**Mid-morning**	4.2 (2.2;7.7)	5.0 (2.9;7.8)	5.8 (3.3;8.0)	.793	.279
**Lunch**	2.6 (1.5;4.3)	2.9 (2.0;4.7)	3.0 (1.8;4.7)	.871	.983
**Afternoon**	2.0 (1.4;2.7)	3.0 (1.7;4.1)	2.4 (1.8;4.6)	.966	.182
**Dinner**	1.4 (0.8;2.5)	1.2 (0.8;1.7)	1.6 (1.0;2.3)	.148	.903
**Bedtime**	1.0 (0.6;1.4)	1.4 (0.7;2.0)	1.1 (0.8;1.9)	.771	.415
**AUCg**	46.9 (36.9;59.4)	47.8 (35.0;54.6)	45.5 (39.8;55.5)	.888	.271

*Note.* AUCg = Area under the curve; CAR = Cortisol Awakening Response; CIRS-CI = Cumulative Illness Rating Scale—Comorbidity Index; MMSE = Mini-Mental State Examination.

We conducted correlation analyses to investigate the associations between baseline cortisol levels and the degree of cognitive impairment. The analyses revealed statistically significant inverse correlations between the MMSE score and cumulative cortisol exposure (AUC; *r* = −0.26, *p* = .039) and afternoon cortisol values (*r* = −0.29, *p* = .026).

### Cortisol trends in the CT-NCD and PH-NCD groups

**Figure 2.**
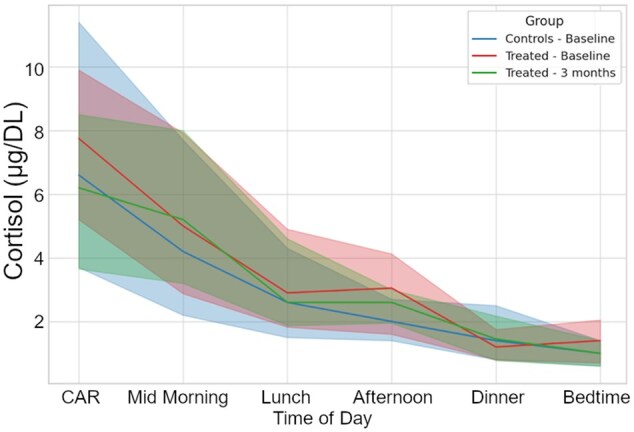
Cortisol daily profile in CT-NCD patients at baseline and after 3 months of cognitive training, compared to healthy controls. CT = cognitive training; CAR = Cortisol Awakening Response; NCD = neurocognitive disorder.

**Figure 3.**
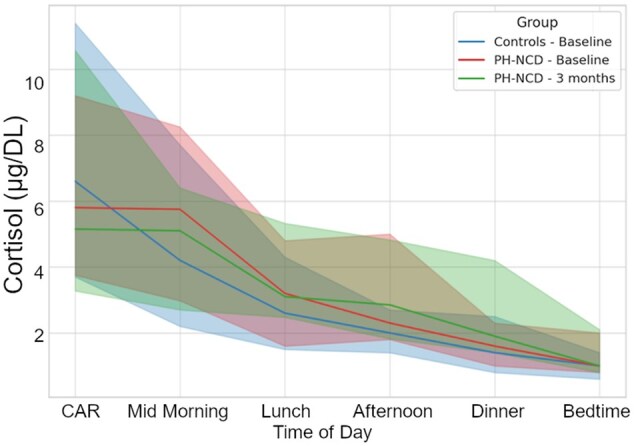
Cortisol daily profile in PH-NCD patients at baseline and after 3 months of pharmacological treatment, compared to healthy controls. CAR = Cortisol Awakening Response; NCD = neurocognitive disorder; PH = pharmacological treatment.

A comparison of the effects of the two different treatments (i.e. drug therapy and cognitive training) on cortisol levels revealed that the CAR significantly decreased after cognitive training [7.75 (5.20; 9.90) vs. 6.20 (3.65; 8.50); *p* = .013], whereas an almost null effect was observed in patients receiving pharmacological therapy (Table 2). Furthermore, before bedtime, cortisol levels showed a trend toward lower levels in the CT-NCD group than in the PH-NCD group after the intervention (*p* = .071). To address baseline group differences, we further compared changes in cortisol levels using ANCOVA models adjusted for baseline cortisol and covariates (age, sex, education, MMSE, comorbidity index). As shown in Supplementary Table 1, the unadjusted analyses indicated significant reductions in CAR and a trend toward a reduction at bedtime in the CT group compared with PH. However, after adjustment, only afternoon cortisol remained significantly different between groups (*F*(1,47)=5.13, *p* = .028, partial η^2^ = 0.099). All other timepoints were no longer statistically significant. Consistently, bivariate analyses did not reveal significant associations between age, sex, education, or comorbidity index with baseline cortisol levels or cortisol changes (all *p* > .05; Supplementary Figure 1). In addition, to further address the potential confounding role of baseline MMSE and education, we conducted an ANCOVA with change in CAR as dependent variable, treatment group as fixed factor, and baseline MMSE, education, comorbidity index, age, and sex as covariates. None of the covariates significantly predicted cortisol change. While the group effect did not reach statistical significance (*F*(1,46) = 1.779, *p* = .189), the direction of the effect was consistent with the unadjusted results, with greater CAR reduction in the CT group (B = –2.418, partial η^2^ = 0.037; Supplementary Table 2). As shown in Figures 2 and 3, both interventions reduced daily cortisol exposure; the decrease was larger in percentage terms in the CT-NCD group (ΔAUC = 17.5% vs. 1.2% in PH), but the between-group difference was not statistically significant (especially after covariate adjustment).

**Table 2.** Comparison of cortisol levels (Nmol/L) at baseline and 3 months after starting the intervention (cognitive training vs. pharmacological therapy).

Variable	CT-NCD			PH-NCD		
	Baseline	3 months	*p*	Baseline	3 months	*p*
**CAR**	7.75 (5.20;9.90)	6.20 (3.65;8.50)	.013	5.80 (3.75;9.20)	5.15 (3.27;10.57)	.533
**Mid-morning**	5.00 (2.87;7.92)	5.20 (3.20;8.00)	.406	5.75 (2.97;8.25)	5.10 (2.70;6.40)	.334
**Lunch**	2.90 (1.82;4.90)	2.60 (1.87;4.60)	.939	3.20 (1.60;4.80)	3.10 (2.47;5.32)	.554
**Afternoon**	3.05 (1.60;4.12)	2.60 (1.95;3.00)	.203	2.30 (1.80;5.00)	2.85 (1.82;4.82)	.841
**Dinner**	1.20 (0.80;1.75)	1.45 (0.67;2.17)	.514	1.60 (1.00;2.30)	1.90 (1.40;4.20)	.122
**Bedtime**	1.40 (0.70;2.05)	1.00 (0.60;1.40)	.071	1.00 (0.80;2.00)	1.00 (0.80;2.10)	.998

*Note.* CAR = cortisol awakening response; CT = training group; NCD = neurocognitive disorder; PH = pharmacological treatment group. Values are expressed as medians (interquartile range).

Additionally, in the CT-NCD group, MMSE scores (mean ± SD) showed a statistically significant improvement over three months (pre-intervention: 23.95 ± 2.71; post-intervention: 25.28 ± 2.28; *p* = .004). In contrast, no significant change was observed in the PH-NCD group (pre-intervention: 20.48 ± 3.98; post-intervention: 20.74 ± 4.30; *p* = .49).

### Six-month trend of cortisol levels in the CT-NCD group

A comparison of cortisol levels at three months with those at six months (Supplementary Figure 2) revealed a tendency toward a further decline in the cortisol levels, but the difference was not significant. The reduction in AUC was confirmed without a significant difference even at six months after training, with a further decrease in cumulative daily cortisol exposure of 6.7%.

## Discussion

The present study highlights the effectiveness of a nonpharmacological cognitive intervention on the levels and patterns of cortisol secretion in patients with neurocognitive disorders. To our knowledge, this is the first study in the literature to directly investigate the effect of cognitive training alone on cortisol levels, contributing to research on the link between neurocognitive disorders and endocrine responses, with an interest in new therapeutic perspectives.

Salivary cortisol levels in patients with NCD were, on average, higher than those in the control group on average across the day, with an overall difference of 16%, in line with a systematic review, which highlighted significantly higher levels of cortisol in the cerebrospinal fluid in subjects with Alzheimer’s Disease (AD) compared to controls (Duarte-Zambrano et al., 2023). In our study, the NCD group, while maintaining a circadian pattern, showed an upward trend in cortisol in the afternoon (2.80 nmol/L in NCD vs. 2.00 nmol/L in controls, *p* = .03). This indicates a selective afternoon elevation consistent with HPA axis dysregulation. This phenomenon, suggestive of a dysregulation of the HPA axis, may have implications for both neurocognitive and behavioral perspectives. Indeed, higher plasma cortisol levels have been associated with greater hippocampal atrophy in MCI patients (White et al., 2023; Wirth et al., 2019). Furthermore, other studies have highlighted that patients with MCI and AD who have higher cortisol levels have an accelerated progression of the disease (Duarte-Zambrano et al., 2023; Ouanes et al., 2022; Udeh-Momoh et al., 2019). From a behavioral point of view, the rise in cortisol in the afternoon could favor the onset of typical behavioral disorders such as sundowning syndrome, which affects more than 25% of patients with neurocognitive disorders (Ferrazzoli et al., 2013; Venturelli et al., 2013). In fact, higher levels of cortisol due to dysregulation of the HPA axis have been linked to neuropsychiatric symptoms that are exacerbated in the late afternoon and evening, contributing to behavioral disturbances at dusk, such as agitation and aggression, as well as worsening of cognitive disorders (Barca et al., 2019; Gerritsen et al., 2022; Menegardo et al., 2019; Todd, 2020). Finally, there was a trend toward higher levels of morning (CAR) and bedtime cortisol in patients with NCD than in controls, consistent with the literature (Barca et al., 2019; Gerritsen et al., 2022; Lovell et al., 2019; Theorell et al., 2021).

Correlation analysis revealed a statistically significant inverse association of afternoon cortisol (and overall AUC) with MMSE scores, whereas the CAR was not significantly associated with MMSE. These findings support the hypothesis that elevated cortisol levels may be associated with worse cognitive function in patients with neurocognitive disorders, which is in line with the studies cited above (White et al., 2023; Wirth et al., 2019). Similarly, higher afternoon cortisol levels have been shown to be associated with poorer cognitive performance across domains, including executive functioning and memory (Ouanes et al., 2022).

With respect to the effects of the two different types of intervention on salivary cortisol levels in patients with NCD, our study indicates that cognitive training showed larger percentage decreases in AUC and reductions in CAR in unadjusted analyses; however, after covariate adjustment, the between-group differences in AUC and CAR were not significant, and only the afternoon time-point remained significant for CT vs. PH. Given that group assignment was clinically determined rather than randomized, these comparisons should be interpreted within the context of baseline cognitive differences between groups. The literature indicates a complex relationship between cortisol levels and cognitive interventions in patients with neurocognitive disorders. Evidence relating to the impact of cognitive interventions on cortisol levels is scarce, and often the effects of non-pharmacological interventions in NCD are measured in terms of improvements in mood and cognitive performance without considering associated biochemical data (Cai et al., 2024; Mao et al., 2021). The only study in this regard is that of Balietti et al. (2023), which, in line with our results, shows a reduction in CAR in patients with AD who underwent an intervention that included cognitive training, psychological support, and socialization activities. However, when we examined both unadjusted and adjusted models side by side, the broader interpretation of these findings became more nuanced. While the unadjusted analyses indicated significant reductions in CAR and a trend at bedtime in the CT group, after adjusting for baseline cortisol, age, sex, education, MMSE, and comorbidity index, only afternoon cortisol remained significantly lower in the CT group compared with the PH group, while CAR and AUC differences were not significant after adjustment. These findings suggest that baseline group differences, particularly in education and MMSE, may have influenced some of the unadjusted results, and underscore the need for cautious interpretation. In contrast, pharmacological treatment did not significantly improve CAR and did not produce significant evening changes. This may reflect differential modulation of the HPA axis by pharmacological approaches, or a more limited influence of AChEIs on endocrine regulation relative to their direct effects on cognition. It should be noted that AChEIs may indirectly modulate stress response and HPA axis activity via central cholinergic pathways, although evidence in humans is limited and mixed (Winek et al., 2021). Memantine, an NMDA receptor antagonist, may influence excitotoxic stress, but its role in cortisol regulation remains unclear. This uncertainty may help explain the modest cortisol changes observed in the PH-group (Hergovich et al., 2001; Réus et al., 2012). The different effectiveness of the two interventions is also evident in the reduction in global exposure to cortisol, which was greater in the CT group (−17.5%) than in the PH group (−1.2%). Our data support the hypothesis that, compared with pharmacological therapy, cognitive training can modulate the HPA axis, with effects most evident in the afternoon. ANCOVA models including baseline MMSE, education, comorbidity, age, and sex, only the afternoon time-point remained significant for CT vs. PH. None of the covariates significantly predicted cortisol change. The group effect on AUC was not significant, although the direction of estimates was consistent with unadjusted analyses. This modulation is complex and likely involves several mechanisms, such as an increase in neuronal plasticity, a protective effect on brain atrophy (through lower exposure to cortisol in brain areas rich in glucocorticoid receptors, such as the hippocampus), and attenuation of the patient’s stress through cognitive engagement. Evidence from both experimental and clinical studies supports the notion that structured cognitive stimulation can reduce allostatic load and promote adaptive neuroendocrine responses. For instance, Manigault et al. (2019) reported that cognitive enrichment in animal models led to reduced circulating cortisol levels and improved cognitive outcomes. These findings are also supported by a systematic review by Da Silva et al. (2021), who demonstrated that cognitive interventions in mature and older individuals not only improve cognitive performance but also provide significant benefits for psychological well-being and quality of life. Several of the included studies reported reductions in anxiety symptoms, enhanced mood, and greater perceived control, underscoring the broader emotional and psychosocial impact of such interventions beyond purely cognitive outcomes. In addition to cognitive stimulation per se, other elements of the CT intervention may have influenced cortisol regulation. Regular social interaction with the trainer and the associated supportive relationship could have reduced stress and promoted neuroendocrine balance. Likewise, the requirement to travel to and from the training site may have led to a modest increase in physical activity, which is also known to modulate HPA axis activity. While these aspects are integral to the CT intervention, they were not matched in the PH group and therefore represent potential confounding factors when interpreting the differential effects of the two treatments.

The analysis six months after cognitive training revealed a downward trend in cortisol levels at critical times of the day, but the difference did not reach significance. However, the further reduction in cumulative daily exposure (−6.7%) suggests that the beneficial effects of the intervention can be maintained over time.

### Limitations and strengths

This study has several limitations. First, the small sample size may have reduced statistical power and limited the generalizability of the findings. This constraint was partly due to the logistical demands of the cognitive training, which required twice-weekly, one-on-one sessions over three months. Second, although most participants were diagnosed with probable Alzheimer’s disease, some heterogeneity in etiology (e.g. mixed dementia) was present. Due to the limited sample size, subgroup analyses based on etiology or diagnosis severity were not feasible. Nonetheless, effects were observed across both treatment groups -CT-NCD and PH-NCD- which included individuals with either mild cognitive impairment (MCI) or mild to moderate major neurocognitive disorder (NCD). We were unable to test for practice effects on MMSE in the healthy control group, since repeated MMSE data were not collected. The significant cortisol reduction observed in the CT group compared to the PH group suggests that the effect is not driven solely by participants with milder impairment. Future studies should examine whether cortisol dysregulation differs across dementia subtypes and severity. Third, individual differences in circadian rhythms and daily stressors may have affected cortisol levels. Nonetheless, several steps were taken to minimize confounding, including strict exclusion criteria and standardized sample collection.

Additionally, it is possible that some of the observed reductions in cortisol, particularly in the CT-NCD group, may reflect regression to the mean rather than a true treatment effect. Given that the CT-NCD group showed slightly higher baseline cortisol levels at certain time points, part of the subsequent decrease may be due to statistical rather than physiological. Although baseline differences were adjusted in the analyses, this phenomenon cannot be completely ruled out in a non-randomized, observational design. Future studies with randomized allocation and larger samples are needed to confirm these findings.

Regarding cognitive training, the paper-and-pencil format used for CS limited the ability to quantify individual performance. Computerized tools could improve precision and allow for dose–response analysis. The lack of data on perceived stress, anxiety, or sense of control limited insight into the psychological impact of the training. Moreover, the adjusted analyses did not show statistically significant group effects. Thus, while the effect size and direction were consistent with the unadjusted results, these findings cannot be considered conclusive and require confirmation in larger randomized studies. Furthermore, when adjusted for baseline cortisol and covariates, most of the between-group differences were no longer significant, with the exception of afternoon cortisol. This indicates that our conclusions regarding the impact of CT on cortisol reduction must be regarded as tentative. Baseline imbalances in education and MMSE may have partially contributed to the unadjusted findings, and future randomized trials with larger samples are needed to clarify these associations.

A major strength of this study is the use of LC–MS/MS, the current gold-standard technique for steroid hormone quantification. Unlike immunoassays, which are susceptible to interference and cross-reactivity, LC–MS/MS ensures greater specificity and accuracy. This is particularly important for salivary cortisol measurements, as immunometric methods may cross-react with cortisone, a structurally similar metabolite that is commonly found at concentrations at least twice those of cortisol in saliva. Moreover, salivary cortisol collection is noninvasive, does not require healthcare personnel, and can be performed at various time points throughout the day, making it a practical and versatile tool suitable for a wide range of research settings and populations.

## Conclusions

The present study suggests that cognitive training may influence the circadian rhythm of cortisol in patients with MCI and mild-to-moderate NCD, who show signs of dysregulation in the HPA axis, including elevated cortisol levels upon awakening and in the afternoon. Although these preliminary findings are promising, they must be interpreted with caution given the small sample size, the non-randomized design, and the exploratory nature of the adjusted analyses. Therefore, the results should not be generalized to all levels of cognitive impairment. Larger randomized studies are needed to confirm and extend these findings.

## Supplementary Material

gbaf243_Supplementary_Data

## Data Availability

The dataset used and analyzed during the current study is available from the corresponding author upon request. This study was not preregistered.
