# Supplementary material for: Effect of cognitive training on cortisol levels in patients with neurocognitive disorders
Source: J Gerontol B Psychol Sci Soc Sci. 2026 Jan 8;81(2):gbaf243. doi: 10.1093/geronb/gbaf243 (PMC12795603; doi:10.1093/geronb/gbaf243)

***The Journals of Gerontology, Series B: Psychological Sciences and Social Sciences* Supplementary Material:** **De Rui et al. Effect of cognitive training on cortisol levels in patients with neurocognitive disorders.**

**Supplementary Table 1.** Between-group effects (CT vs PH) on cortisol changes after 3 months: unadjusted vs adjusted models.

| **Cortisol outcome** | ***p* (unadjusted)*** | **F (df) unadjusted** | ***p* (adjusted)**** | **F (df) adjusted** | **Partial η² (adjusted)** |
| --- | --- | --- | --- | --- | --- |
| CAR | 0.013 | — | 0.602 | F(1,45)=0.276 | 0.006 |
| Mid-morning | 0.406 | — | 0.291 | F(1,49)=1.139 | 0.023 |
| Lunch | 0.939 | — | 0.754 | F(1,48)=0.099 | 0.002 |
| Afternoon | 0.203 | — | 0.028* | F(1,47)=5.134 | 0.099 |
| Dinner | 0.514 | — | 0.522 | F(1,50)=0.415 | 0.008 |
| Bedtime | 0.071 | — | 0.772 | F(1,49)=0.085 | 0.002 |

* Unadjusted *p*-values are from within-group paired comparisons (baseline vs 3 months, see Table 2) and between-group non-parametric tests.

** Adjusted models (ANCOVA) include group (CT vs PH) as a fixed factor, and baseline cortisol, age, sex, education, MMSE, and the comorbidity index (CIRS) as covariates. Partial η² values were calculated as SS_effect / (SS_effect + SS_error).

* *p* < 0.05.

**Supplementary Table 2.** ANCOVA on CAR change (ΔCAR) between CT-NCD and PH-NCD

| **Source** | **B (unstandardized)** | **F (df)** | ***p*** | **Partial η²** |
| --- | --- | --- | --- | --- |
| **Group (CT vs PH)** | –2.418 | F(1,46)=1.779 | 0.189 | 0.037 |
| **Age** | — | <1 | >0.2 | — |
| **Sex** | — | <1 | >0.2 | — |
| **Education** | — | <1 | >0.2 | — |
| **MMSE** | — | <1 | >0.2 | — |
| **CIRS** | — | <1 | >0.2 | — |

Note. CAR = Cortisol Awakening Response. ANCOVA model with delta_CAR as dependent variable, treatment group as fixed factor, and baseline MMSE, education, comorbidity index (CIRS), age, and sex as covariates. None of the covariates was a statistically significant predictor.

**Supplementary Figure 1.** Correlation matrices (a) between baseline covariates and cortisol levels and (b) between baseline covariates and cortisol changes at 3 months. Pearson correlation coefficients (r) are shown with corresponding *p*-values in each cell.

**
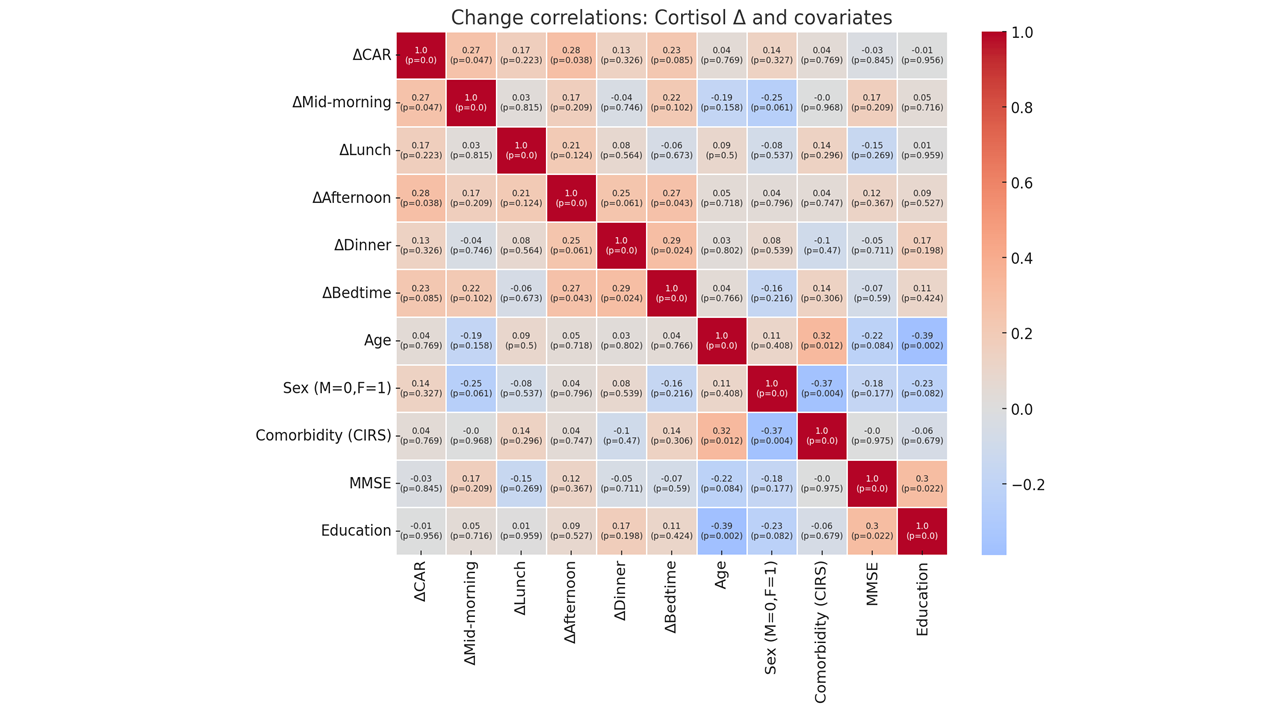
**

**Supplementary Figure 2**. Cortisol trends in CT-NCD patients at baseline, 3 months, and 6 months vs. controls.

Note: CAR = Cortisol Awakening Response.


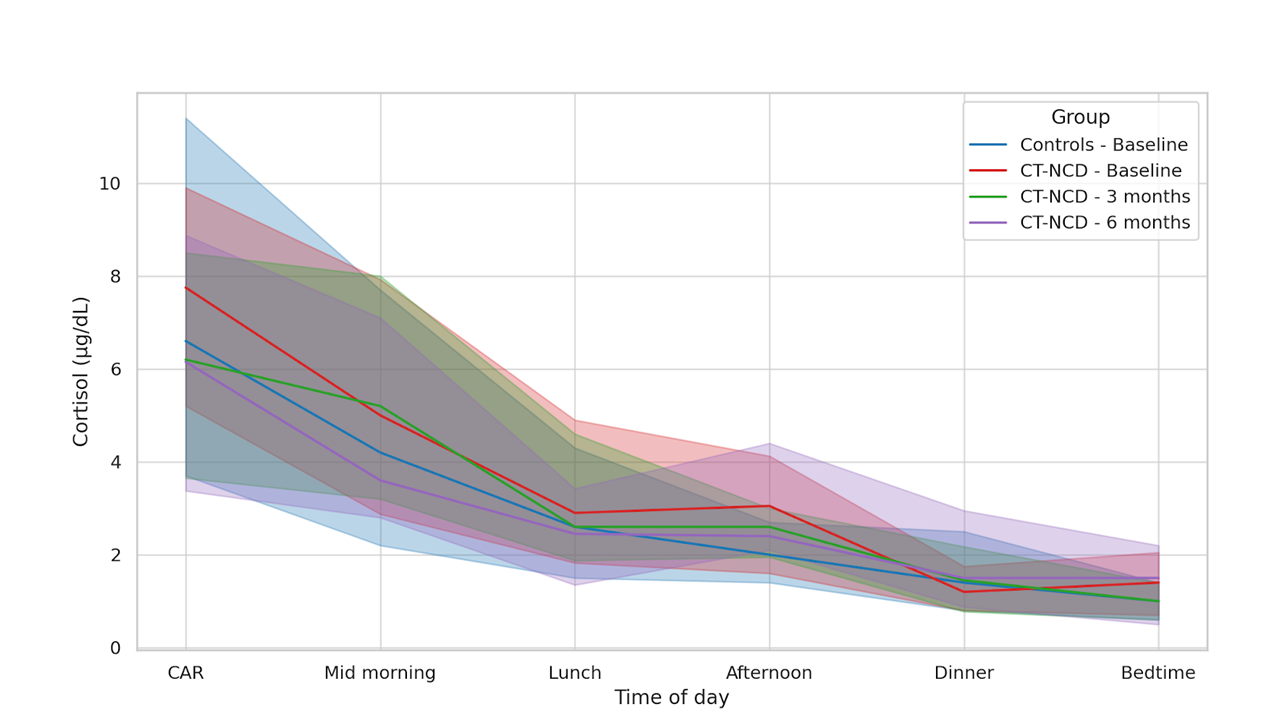

Supplement: gbaf243_Supplementary_Data [file gbaf243_supplementary_data.zip › JGPS suppl De Rui et al.docx]
