# Supplementary figures and images for: Effect of cognitive training on cortisol levels in patients with neurocognitive disorders
Source: J Gerontol B Psychol Sci Soc Sci. 2026 Jan 8;81(2):gbaf243. doi: 10.1093/geronb/gbaf243 (PMC12795603; doi:10.1093/geronb/gbaf243)

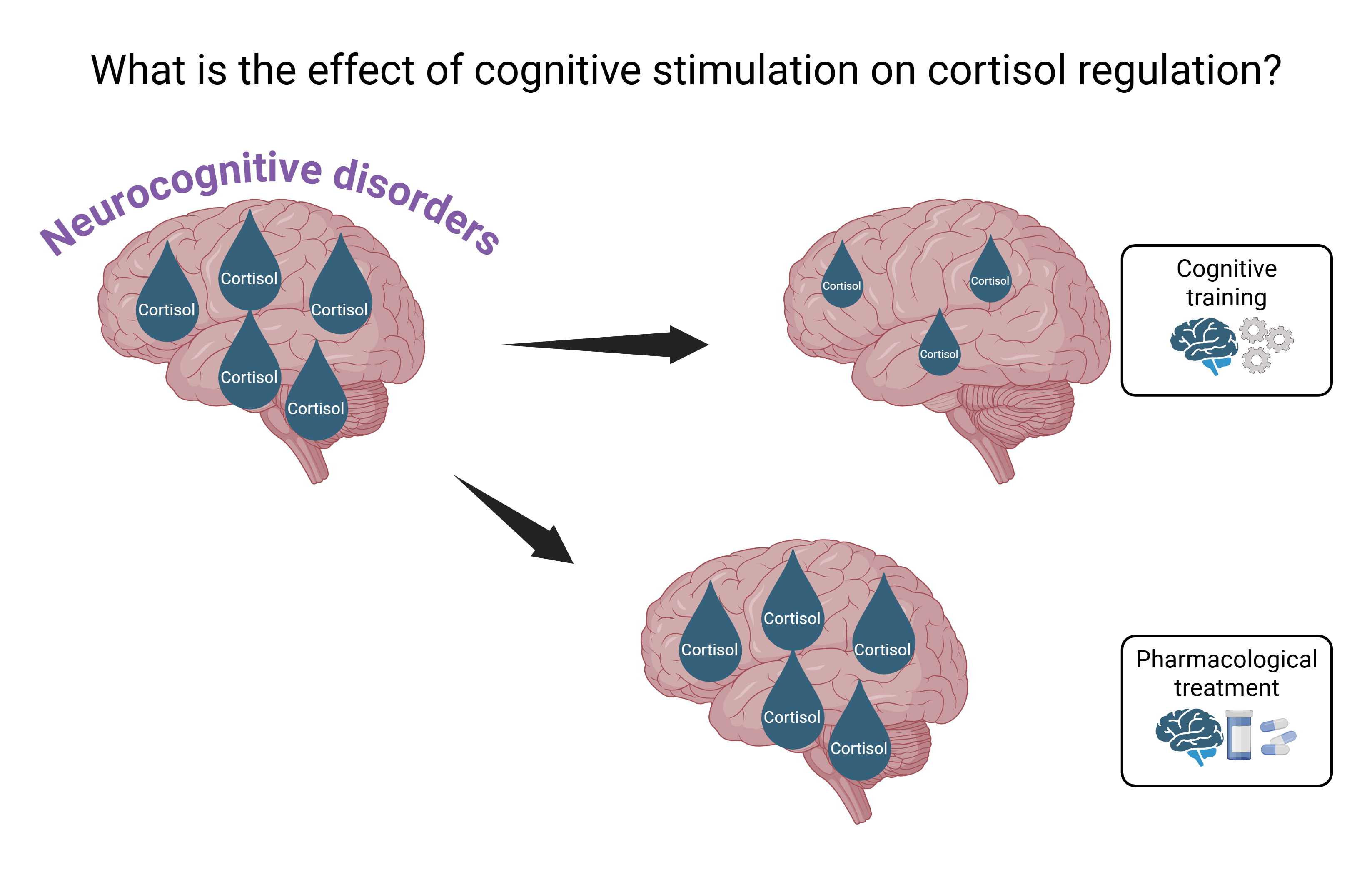

Supplement: gbaf243_Supplementary_Data [file gbaf243_supplementary_data.zip › graphical_abstract.png]
